# Supplementary material for: Comparative restriction enzyme analysis of methylation (CREAM) reveals methylome variability within a clonal in vitro cannabis population
Source: Front Plant Sci. 2024 May 30;15:1381154. doi: 10.3389/fpls.2024.1381154 (PMC11169872; doi:10.3389/fpls.2024.1381154)
Supplement: Supplementary file 2 [file DataSheet_2.pdf]

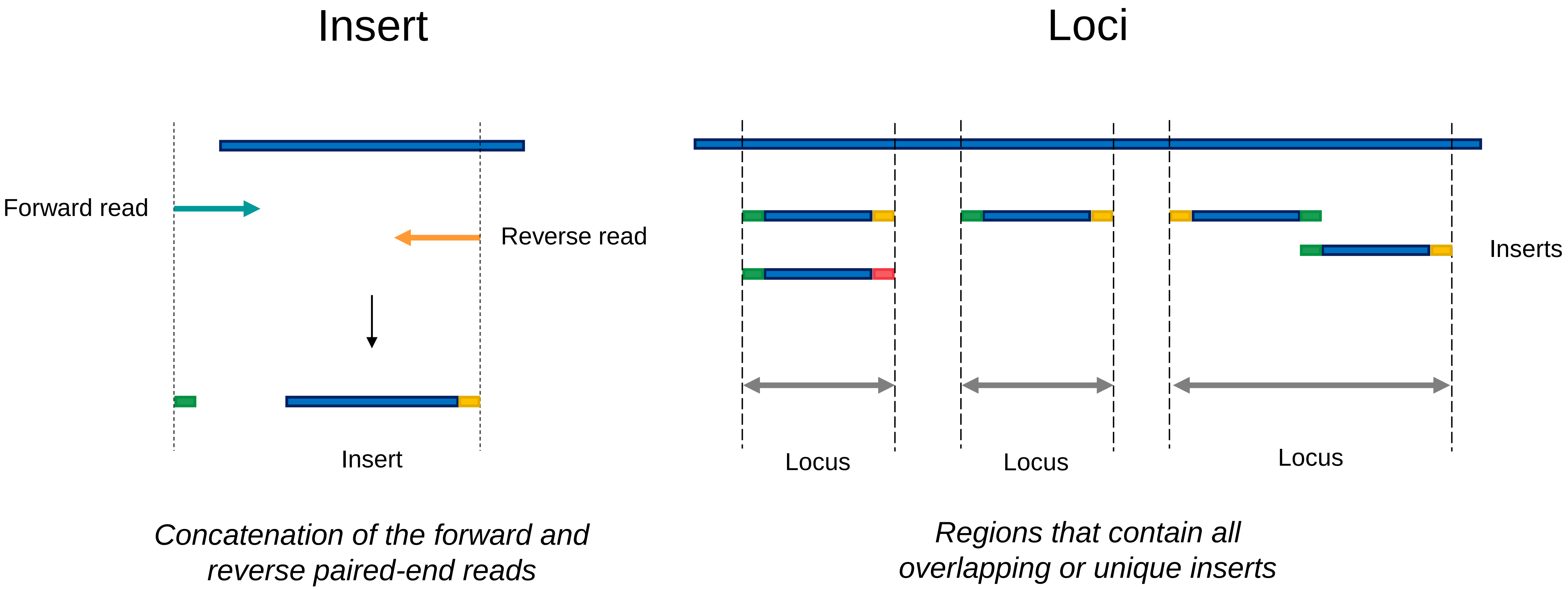

Supplementary Figure 2. Visual representation of inserts and loci as defined in the context of this study.
