# Supplementary material for: Comparative restriction enzyme analysis of methylation (CREAM) reveals methylome variability within a clonal in vitro cannabis population
Source: Front Plant Sci. 2024 May 30;15:1381154. doi: 10.3389/fpls.2024.1381154 (PMC11169872; doi:10.3389/fpls.2024.1381154)
Supplement: Supplementary file 3 [file DataSheet_3.pdf]

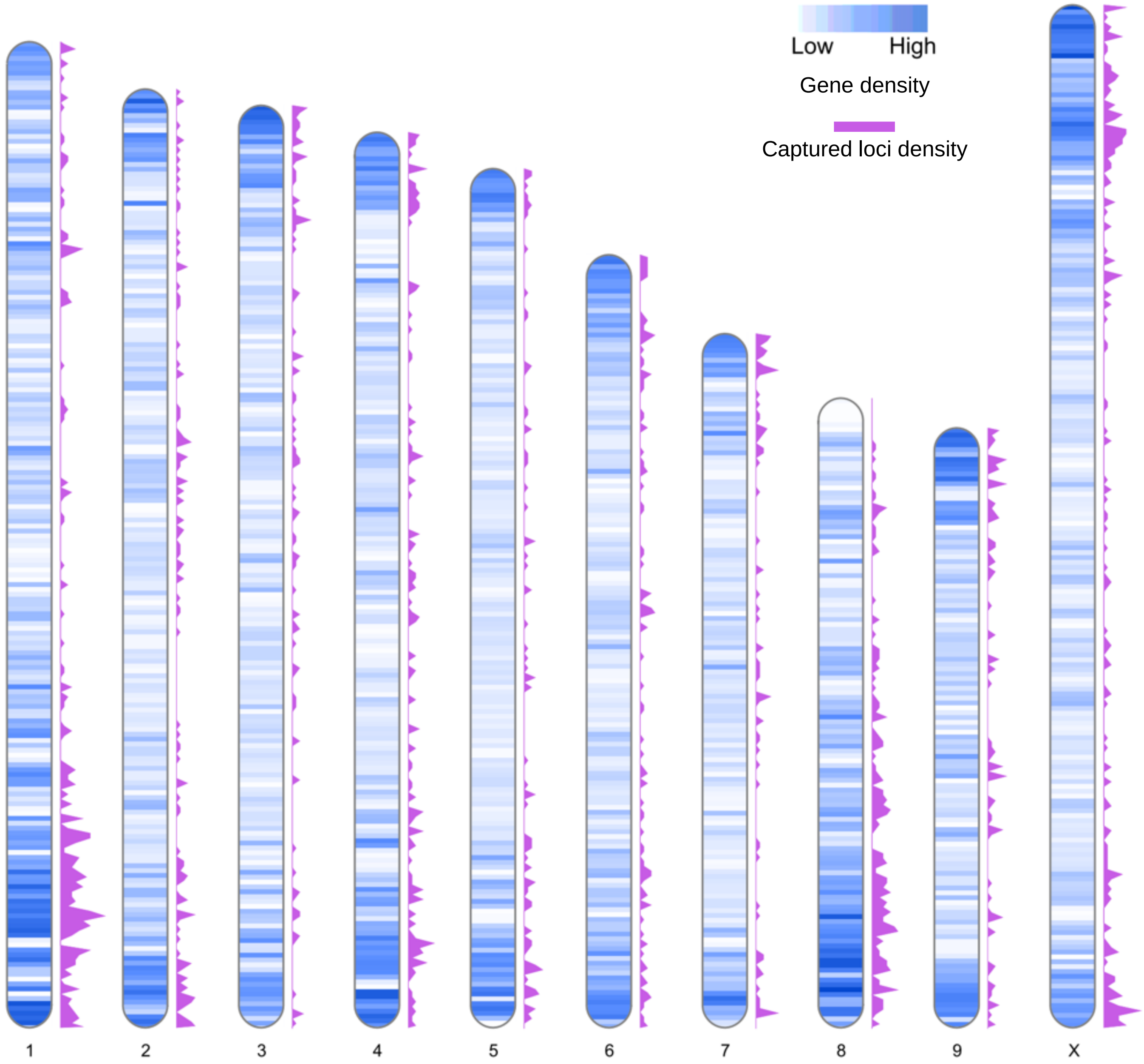

Supplementary Figure 3. Density of genes and the density of captured loci with the CREAM approach on the chromosomes of the cs10 cannabis reference genome in bins of 500 kb. Spearman's rank correlation coefficient = 0.6913.
