# Supplementary material for: Comparative restriction enzyme analysis of methylation (CREAM) reveals methylome variability within a clonal in vitro cannabis population
Source: Front Plant Sci. 2024 May 30;15:1381154. doi: 10.3389/fpls.2024.1381154 (PMC11169872; doi:10.3389/fpls.2024.1381154)
Supplement: Supplementary file 4 [file Table_1.docx]

Supplementary Material

Comparative Restriction Enzyme Analysis of Methylation (CREAM) Reveals Methylome Variability Within a Clonal *In Vitro* Cannabis Population

**Justin Boissinot^1,2,3,4^, Kristian Adamek^5^, Andrew Maxwell Phineas Jones^5^, Eric Normandeau^2^, Brian Boyle^2^, Davoud Torkamaneh^1,2,3,4^***

*** Correspondence:** Davoud Torkamaneh: [davoud.torkamaneh.1@ulaval.ca](mailto:davoud.torkamaneh.1@ulaval.ca)

**Supplementary Table 1.** Genome coverage (%) and mean depth of coverage (X) for each CREAM library.

|  | **Genome coverage (%)** | | | **Mean depth of coverage (X)** | | |
| --- | --- | --- | --- | --- | --- | --- |
|  | ***Msp*I** | ***Hpa*II** | **Overall** | ***Msp*I** | ***Hpa*II** | **Overall** |
| **Mean** | 0.488 | 0.286 | 0.387 | 76.654 | 114.147 | 95.401 |
| **Max** | 0.885 | 0.629 | 0.885 | 164.033 | 231.384 | 231.384 |
| **Min** | 0.206 | 0.088 | 0.088 | 17.694 | 21.300 | 17.694 |
