# Supplementary material for: Comparative restriction enzyme analysis of methylation (CREAM) reveals methylome variability within a clonal in vitro cannabis population
Source: Front Plant Sci. 2024 May 30;15:1381154. doi: 10.3389/fpls.2024.1381154 (PMC11169872; doi:10.3389/fpls.2024.1381154)
Supplement: Supplementary file 1 [file DataSheet_1.pdf]

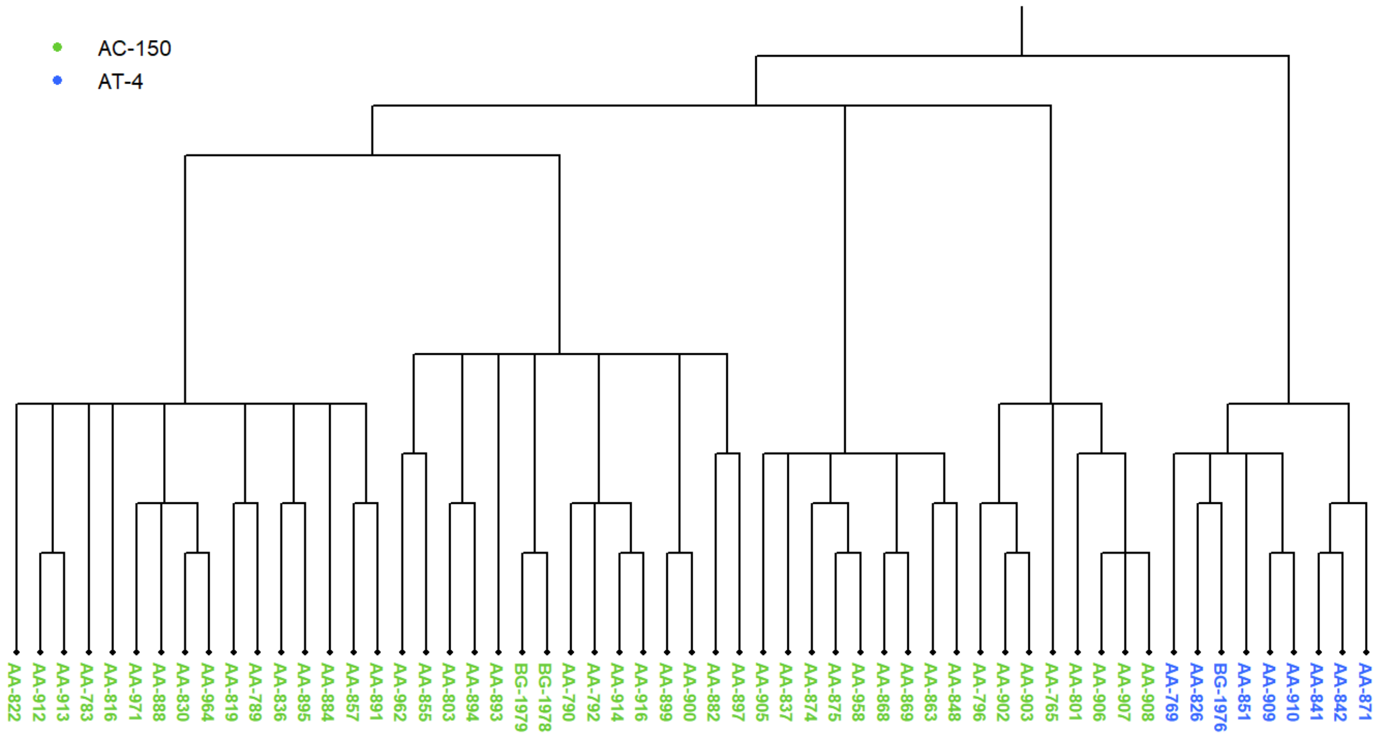

Supplementary Figure 1. Population of cannabis clones used in this study. Clonal lines are represented in green (AC-150) and blue (AT-4). Population tree was loaded from an XML file and generated using the *rphyloxml* and *ape* R packages, respectively.
